# Development and application of an indirect ELISA to detect antibodies to *Neospora caninum* in cattle based on a chimeric protein rSRS2-SAG1-GRA7

**DOI:** 10.3389/fvets.2022.1028677

**Published:** 2022-12-15

**Authors:** Cong-Shan Yang, Chuan-Yin Yang, Olalekan-Opeyemi Ayanniyi, Ya-Qian Chen, Zhen-Xiao Lu, Jin-Yi Zhang, Lu-Yao Liu, Yu-Hang Hong, Rong-Rong Cheng, Xiang Zhang, Qin-Qin Zong, Hong-Xi Zhao, Qian-Ming Xu

**Affiliations:** ^1^Department of Veterinary Medicine, College of Animal Science and Technology, Anhui Agricultural University, Hefei, Anhui, China; ^2^Department of Veterinary Medicine, School of Agriculture, Ningxia University, Yinchuan, China

**Keywords:** *Neospora caninum*, chimeric protein, ELISA, abortion, dairy cattle, Ningxia, seroprevalence

## Abstract

*Neospora caninum* is an important apicomplexan parasite causing neosporosis in cattle. The disease is recognized as one of the most important cause of reproductive problems and abortion in cattle worldwide. In this context, we developed an indirect enzyme-linked immunosorbent assays (ELISA) with chimeric protein rSRS2-SAG1-GRA7 to diagnose antibodies to *Neospora*-infection. This indirect ELISA was compared to indirect fluorescent antibody test (IFAT) and western blotting (WB), and the sensitivity and specificity results of ELISA were calculated to be 86.7 and 96.1%, respectively. The overall coincidence rate was 92.6% using IFAT and WB. Additionally, 329 aborting dairy cattle serum samples were tested using this ELISA to evaluate the prevalence of *N. caninum* in Ningxia, China. The positive rate of *N. caninum* in these farms was from 19.05 to 57.89%, and the mean rate was 41.64% (±11.01%), indicating that infection with *N. caninum* may be one of the important causes of cattle abortion in this region. This established rSRS2-SAG1-GRA7 indirect ELISA is capable for detecting the antibodies against *N. caninum*, and it could be a useful screening tool for monitoring the epidemiology of neosporosis in cattle.

## 1. Introduction

*Neospora caninum* (Apicomplexa: Coccidia) is the etiologic agent of neosporosis (1). This parasite was initially recognized in 1984 in dogs from Norway (2). Dogs and other related canids are the definitive host of *N. caninum*, while many mammals such as cattle and sheep are intermediate hosts (3). Neosporosis is recognized as one of the most important causes of reproductive problems especially abortion in cattle affecting the development of the cattle and dairy industries worldwide (4, 5). The abortion in cattle can occur starting from third month of gestation until delivery (6–8). *N. caninum* can also cause fetal viability disorders or neurological birth defects in newborn calves and those younger than 2 months of age (9–11). Cattle can become infected horizontally *via* the ingestion of contaminated food by sporulated oocysts or vertically by the transmission of the parasite from the dam to the fetus, which is considered the main route of infection in cattle (11).

The indirect fluorescent antibody test (IFAT) is a “gold standard” to detect antibodies in *N. caninum* infection (3). With the development of large-scale epidemiological research, the ELISA testing method is widely used, and it is gradually becoming the major method of serological testing (12). Compared to IFAT, ELISA has a relatively simple procedure, and the results determination is not affected by subject factors, therefore it is more suitable for serological and epidemiological investigations of large quantities of serum samples (13).

The surface proteins of *N. caninum* NcSRS2 and NcSAG1 are immunodominant antigens that play a key role in the host cell recognition, adhesion, and invasion (14, 15). Jenkins et al. (16) investigated the immune effect of plasmid DNA encoding NcGRA6 and NcGRA7, and confirmed that NcGRA7 can be used as an immunogen in the prevention of neosporosis (16). The NcSRS2 protein is expressed in tachyzoites and bradyzoites, with a 44% sequence identity to *T. gondii* TgSRS2, the NcSAG1 protein is one of the major tachyzoite surface protein, and displays 53% identity to *T. gondii* TgSAG1, and the dense granule protein NcGRA7 is with a 42% sequence identity to *T. gondii* TgGRA7 (17–19). The sensitivity and specificity of indirect ELISA based on NcSRS2, NcSAG1, or NcGRA7 have been studied, and Alves et al. (20) confirmed that sensitivity and specificity of indirect ELISA performed with the proteins NcSRS2 and NcSAG1 in combination had higher than individually (20). However, the chimeric expression of these three proteins has not been studied.

In this study, we aimed to establish an indirect ELISA based upon chimeric protein rSRS2-SAG1-GRA7 in the prokaryotic expression system for assessment of *N. caninum* seroprevalence in aborting dairy cattle from Ningxia province of China.

## 2. Materials and methods

### 2.1. Ethical statement

This study was performed in accordance with the recommendations in the Guide for the Care and Use of Laboratory Animals of the Ministry of Health, China. The study was approved by the Institutional Animal Care and Use Committee of Anhui Agricultural University (No. AHAU2020020). This study was carried out in compliance with the ARRIVE guidelines. In addition, all the samplings was done when the informed consent was obtained from farm owners.

### 2.2. Study design

The genes coding for the NcSRS2, NcSAG1 and NcGRA7 proteins of *N. caninum* were cloned and expressed in pET-28a expression vector. The indirect ELISA was developed using the purified recombinant protein and the results were compared with IFA and WB by evaluating 81 sera samples. Additionally, 329 serum samples from aborting dairy cattle in Ningxia were examined for *N. caninum* infections by using indirect ELISA.

### 2.3. Parasites and cell cultures

African Green Monkey kidney cell (Vero) lines were cultured in Dulbecco's Modified Eagle's Medium (DMEM) containing 25 mM glucose and 4 mM glutamine and supplemented with 10% fetal bovine serum (FBS, Gibco, USA) as previously described (21). *N. caninum* tachyzoites were maintained *in vitro* by serial passages on confluent Vero cells. Cells and parasites were incubated at 37°C with 5% CO_2_ in a humidified incubator.

### 2.4. Sampling

**Figure 1:**
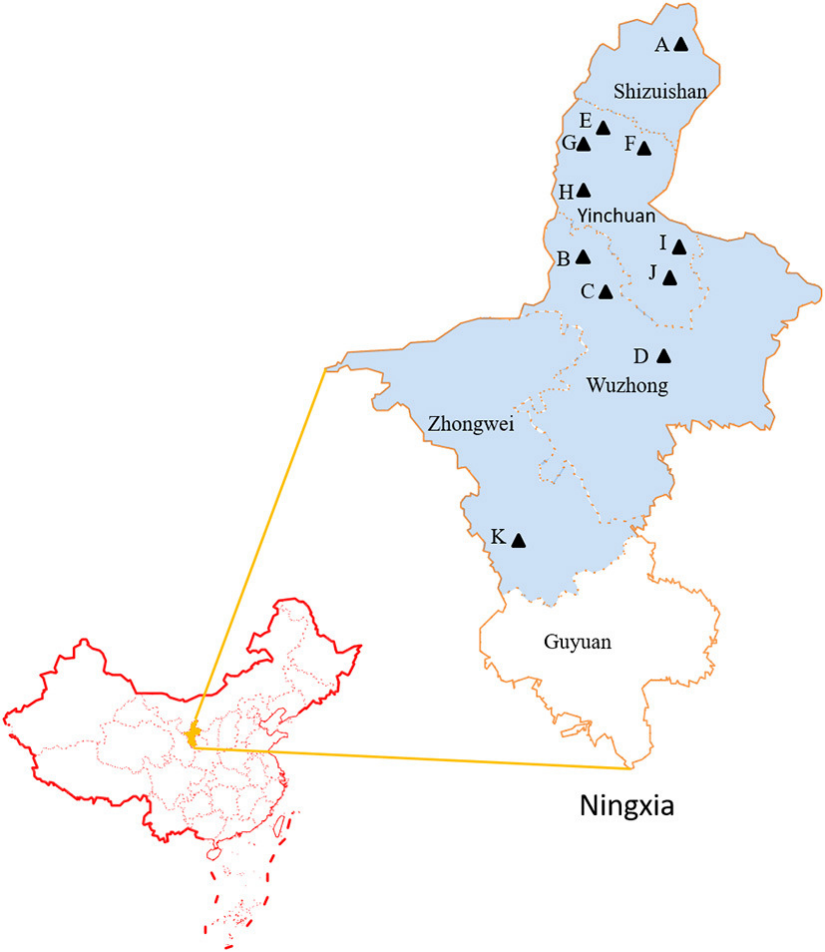
The geographic map of surveyed cities located in Ningxia of China. **(A-K)** The samples collected areas are indicated with black triangle.

A total of 329 serum samples used to evaluate the prevalence of *N. caninum* in Ningxia of China were collected between 2017 and 2021 from aborting dairy cattle that were the breeding adult cows suffered abortion in 11 farms located in Ningxia with approximately 450, 000 cows (Figure 1), and sera were collected within 3 days after the abortion occurred. The farms were located in 4 cities: Shizuishan (No. = 1), Wuzhong (No. = 3), Yinchuan (No. = 6), Zhongwei (No. = 1) where *N. caninum* was endemic (22–25). Out of the 81 sera samples used to evaluate indirect ELISA, 53 were collected from non-aborting cattle in Anhui Province, and the remaining 13 positive and 15 negative field sera samples tested by IFAT and WB were generously provided by Prof. Qun Liu of China Agricultural University. These sera were separated by centrifugation at 1500 × g for 10 min and stored at −20°C until use. A pool containing sera of 10 naturally infected cattle with immune-reactivity against *N. caninum* antigens in IFAT and WB was used as the positive control. Sera collected from newborn calf that did not present reproductive problems and analyzed by IFAT and WB with *N. caninum* negativity was used as the negative control. Fifty serum samples collected from cattle in Gansu Province that were confirmed negative for neosporosis by IFAT and WB were used to determine the cut-off value of indirect ELISA. The positive control sera, negative control sera and 50 sera negative samples were preserved in Animal Parasites and Parasitic Epidemiology Laboratory, Anhui Agricultural University, China.

### 2.5. Construction of the chimeric plasmid

All primer sequences are shown in Table 1. *N. caninum* NcSRS2, NcSAG1 and NcGRA7 (GenBank: AF061249, AF132217 and AF176649) genes were amplified using primers P1 and P2, P3 and P4, and P5 and P6 respectively. The expression plasmid backbone was amplified using primers P-up and P-down with plasmid pET-28a as template. The NcSRS2 and NcSAG1 genes were sequenced and ligated into the pET-28a backbone vector using the Basic Assembly Mix (TransGen Biotech; Beijing, China) according to the manufacturer's protocol, the resulting chimeric expression plasmid was named 28a-S2-G1. Then the 28a-S2-G1 carrier skeleton was amplified using primers P-up and P4-2 with plasmid 28a-S2-G1 as template, and the NcGRA7 gene was sequenced and ligated with the 28a-S2-G1 carrier skeleton, the resulting chimeric expression plasmid was named 28a-S2-G1-A7. The linker1 was added to the 5' ends of P2 and P3, and the linker2 was added to the 5' ends of P4-2 and P5, respectively.

**Table 1:** Primers for amplifying the NcSRS2, NcSAG1, NcGRA7, pET-28a plasmid backbone and 28a-S2-G1 carrier skeleton, and product sizes.

**Primers**	**Sequences (5' to 3')**	**Product size (bp)**	**Target gene**
P1	GGGTCGCGGATCCGAATTCGCGCCGTTCAAGTCGGAAAA	629	*NcSRS2*
P2	CATCTCCCGCCAACTTGAGGAGGTCGAAGTTCAGAGAATAAGGGCGTTCTTTACATT		
P3	CTCAAGTTGGCGGGAGATGTTGAGTCCAACCCTGGGCCCACCTGTGACAACGAAGAGAA	565	*NcSAG1*
P4	GGTGGTGGTGGTGCTCGAGCTGCTTAGGATCAGGAGGAA		
P5	CAAGGGCTCGGGCTCGACCCAGCTGGCTAGCGCTGGAGACTTGGC	257	*NcGRA7*
P6	GGTGGTGGTGGTGCTCGAGGATCAAGCTGCCTTTCTGAG		
P-up	CTCGAGCACCACCACCACCACCAC	5340	pET-28a backbone
P-down	GAATTCGGATCCGCGACCCATTTGC		
P4-2	GTCGAGCCCGAGCCCTTGCTAGCCTGCTTAGGATCAGGAGG	6500	28a-S2-G1 skeleton
P-up	CTCGAGCACCACCACCACCACCAC		

The chimeric expression plasmid 28a-S2-G1-A7 transformed into a strain of competent *E. coli* cells, DH5α. The transformed colonies were selected from Luria-Bertani (LB) agar (Solarbio; Beijing, China) plates containing kanamycin (50 μg/mL) and were identified by PCR. Subsequently, the chimeric expression plasmid 28a-S2-G1-A7 was transformed into *E. coli* Rosseta.

### 2.6. Expression and purification of the chimeric protein rSRS2-SAG1-GRA7

The positive transformants were cultured in LB medium (Solarbio; Beijing, China) containing 50 μg/mL kanamycin with vigorous shaking at 37 °C until the 600 nm optical density (OD) of bacteria cultures reached approximately 0.4–0.6, were induced chemically by using 1 mM isopropyl-β-D-thiogalactoside (IPTG) for 5 h. The expression of chimeric proteins was analyzed by 12% (v/v) sodium dodecyl sulfate-polyacrylamide gel electrophoresis (SDS-PAGE) and the gels were stained with coomassie brilliant blue. Induced cells were washed in phosphate-buffered saline (PBS) two times, then lysed by sonication in an ice-water bath. The lysed cells were centrifuged at 10,000 g for 15 min. The supernatant and the precipitate were then subjected to SDS-PAGE analysis. The chimeric protein was mainly expressed as insoluble form. The inclusion body was dissolved in 8 M urea solution and purified by affinity chromatography on a HisTrap™ Sepharose nickel column (Beyotime Biotechnology; Shanghai, China). And the concentration was determined using a BCA kit (Beyotime Biotechnology; Shanghai, China) as previously described (20).

### 2.7. Western blotting

Western blotting to detect the cross-reactivity with *T. gondii* of chimeric protein rSRS2-SAG1-GRA7 was performed as described previously (26). Purified chimeric protein was subjected to 12% (v/v) SDS-PAGE, and the gel was prepared for WB as follows. Chimeric proteins separated in the gel were electrically transferred to a PVDF membrane and the membranes were blocked 1 h at room temperature with phosphate-buffered saline which contained 5% (w/v) skimmed milk powder. The membrane was then incubated with mouse serum positive for neosporosis and mouse serum positive for toxoplasmosis for 1 h at room temperature on a plate shaker. The mouse serum was performed as described previously (26). Following this incubation, the membrane was washed three times with PBS buffer and reacted with HRP-goat anti-mouse IgG (1:1000 dilution in PBS) at room temperature for 1 h. After three washes, the reactive bands were visualized using enhanced chemiluminescence reagents (Beyotime Biotechnology; Shanghai, China).

Detection of specific *N. caninum* antibodies was carried out by WB as described previously (27). Tachyzoites of NC-1 isolate were used for antigen preparation under non-reducing conditions. Bovine serum samples were diluted 1:100. A HRP-conjugated anti-cattle total IgG (1:1000 dilution in PBS) (Solarbio; Beijing, China) was used. The WB process and reactive bands visualization were performed as described above.

### 2.8. Indirect fluorescent antibody test (IFAT)

The antigen was produced by culturing tachyzoites of *N. caninum* Nc1 strain as described previously (28). 1 × 10^5^ *N. caninum* tachyzoites were spotted on slides in 12-well plates and allowed to air-dry overnight. All slides were fixed with formaldehyde and stored at −20°C until used. The IFAT procedures were performed as described previously with some modifications (29). Each serum sample was diluted at 1:50 using sterile saline solution buffered by phosphate (PBS), pH of 7.0, for screening. 20 μL of each sera samples were placed on slides and the slides were incubated at 37°C for 30 min in a box containing moisture content 100%. The slides were washed three times in a sterile carbonated buffer, pH of 9.0 with 5 min for each washing. Fluorescein isothiocyanate (FITC) conjugated goat anti-bovine IgG (Solarbio; Beijing, China) diluted at 1:100 was added to each slide before the slides were incubated again at 37°C for 30 min in a box containing moisture content 100%. The slides were washed in PBS, as was described earlier, and mounted in buffered glycerin (pH of 8.0). Observation of complete peripheral fluorescence of the tachyzoites was considered a positive result (28).

### 2.9. Chimeric protein rSRS2-SAG1-GRA7 indirect ELISA

Polystyrene 96-well microplates (NEST; Wuxi, China) were coated with chimeric protein rSRS2-SAG1-GRA7 diluted in carbonate buffer (pH 9.6) at a final concentration of 0.5 μg/ml, and plates incubated for 1 h at 37 °C. After five washes with PBST (PBS containing 0.05% Tween 20), the plates were blocked by PBST plus 5 % skimmed milk powder (SMP) for 2 h at 37 °C. Then, the plates were washed five times with PBST, and 100 μL of sera sample diluted at 1:100 in serum dilutions (10 mM PBS, 0.1% Tween 20, 0.2% Fish gelatin and 0.3% BSA) (Baiditai; Jining, China) was added in duplicate. After 1 h of incubation at 37 °C and five washes with PBST, 100 μL of HRP-conjugated anti-cattle total IgG (Solarbio; Beijing, China) diluted at 1:3000 in PBST, were added to the plates for 1 h at 37 °C. The reaction was developed with 100 μL of TMB coloring solution, at 37 °C in the dark for 20 min, TMB coloring solution (Solarbio; Beijing, China) becomes blue color at HRP-conjugated anti-cattle. The reaction was terminated by 50 μL of stop solution. Subsequently, the OD value was measured at a wavelength of 450 nm using a microplate reader (Bio-Rad; Hercules, USA). ELISA was performed in duplicate for all samples, with three positive and three negative controls on each plate. The OD value of the blank was automatically subtracted from each sample value.

### 2.10. Determination of the cut-off value

Fifty serum samples of cattle were collected to determine the cut-off value of this assay. These serum samples were confirmed negative for neosporosis by IFAT and WB described, and then were used to define the cut-off value in the rSRS2-SAG1-GRA7 indirect ELISA. The OD450 value of these cattle serum negative for neosporosis obtained in this rSRS2-SAG1-GRA7 indirect ELISA were defined to calculate the cut-off value. The mean OD450 value + 3 × standard deviations was defined as the cut-off value. OD450 value of samples greater than or equal to the cut-off value were considered positive serum for neosporosis.

### 2.11. Statistical analysis

A total of 81 serum samples of cattle tested using this ELISA method, the results obtained from the sera samples were subjected to receiver operating characteristic (ROC) analysis using the MedCalc statistical software (version 19.5.6). As a comparison, IFAT and WB were applied to test these samples and act as reference methods to distinguish positive or negative samples. The results of the three methods were compared, and the sensitivity and specificity of detection were calculated to evaluate the accuracy of the indirect ELISA. All other statistical data were analyzed using GraphPad Prism 5 (version 5.01; GraphPad Software, San Diego, CA, USA). The results were expressed as mean ± SD and evaluated by non-parametric tests. Values of *P* < 0.05 were considered statistically significant.

## 3. Results

### 3.1. Expression and purification of chimeric protein rSRS2-SAG1-GRA7

**Figure 2:**
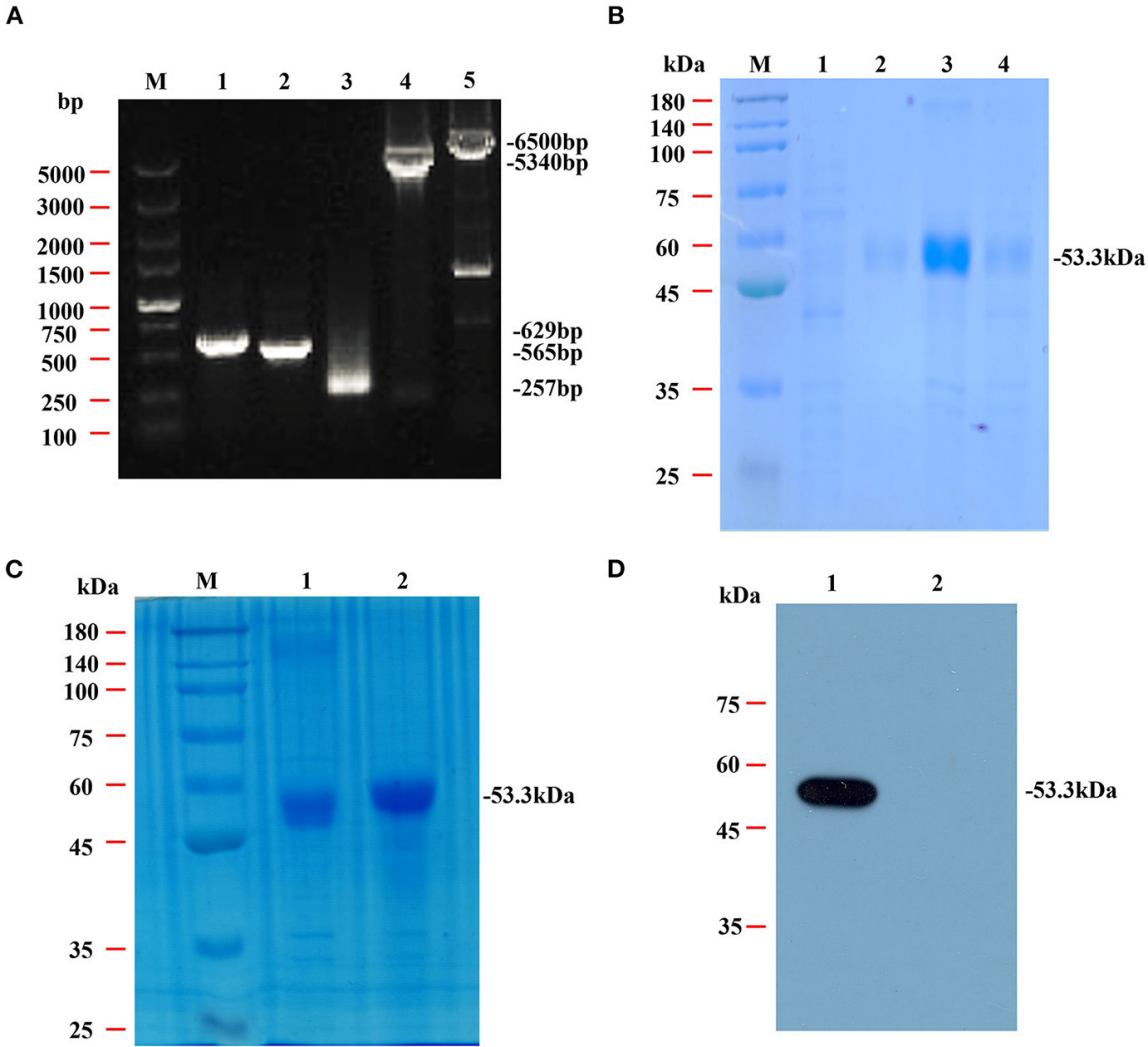
Amplification, SDS-PAGE and western blotting analysis of the chimeric protein rSRS2-SAG1-GRA7. **(A)** PCR amplification of the NcSRS2, NcSAG1, NcGRA7 genes and plasmid backbone fragment. Lane M, DL5000 DNA marker. Lane 1, the NcSRS2 gene fragment. Lane 2, the NcSAG1 gene fragment. Lane 3, the NcGRA7 gene fragment. Lane 4, the pET-28a plasmid backbone fragment. Lane 5, the 28a-S2-G1 carrier skeleton fragment. **(B)** SDS-PAGE analysis of chimeric protein rSRS2-SAG1-GRA7. Lane M, prestained protein molecular weight standard. Lane 1, uninduced *E. coli* cell lysate. Lane 2, the supernatant of induced *E. coli* cell lysate. Lane 3, the precipitate of induced *E. coli* cell lysate. Lane 4, induced *E. coli* cell lysate. **(C)** SDS-PAGE analysis of chimeric protein rSRS2-SAG1-GRA7. Lane M, prestained protein molecular weight standard. Lane 1, induced *E. coli* cell lysate. Lane 2, purified chimeric protein rSRS2-SAG1-GRA7 by affinity chromatography of Ni-NTA spin column. **(D)** Western blotting analysis of chimeric protein rSRS2-SAG1-GRA7. Lane 1, Chimeric protein rSRS2-SAG1-GRA7 and mouse serum positive for neosporosis. Lane 2, Chimeric protein rSRS2-SAG1-GRA7 and mouse serum positive for toxoplasmosis.

The genes for NcSRS2, NcSAG1 and NcGRA7 were amplified by PCR (Figure 2A) and cloned into a pET-28 system, and the inserted genes were sequenced to ensure the correctness of the reading frame. As shown by SDS-PAGE (Figure 2B), the chimeric protein rSRS2-SAG1-GRA7 was expressed as soluble and insoluble forms, up to 90% of all proteins are insoluble, and the molecular mass of the chimeric protein was estimated at approximately 53 kDa, which was in agreement with that deduced from its amino acid sequence (Figure 2C). The reactivity of the purified chimeric protein was detected by WB. The result revealed that the chimeric protein was specifically bound by mouse serum positive for neosporosis, but unbound by mouse serum positive for toxoplasmosis (Figure 2D).

### 3.2. rSRS2-SAG1-GRA7 indirect ELISA validation

**Figure 3:**
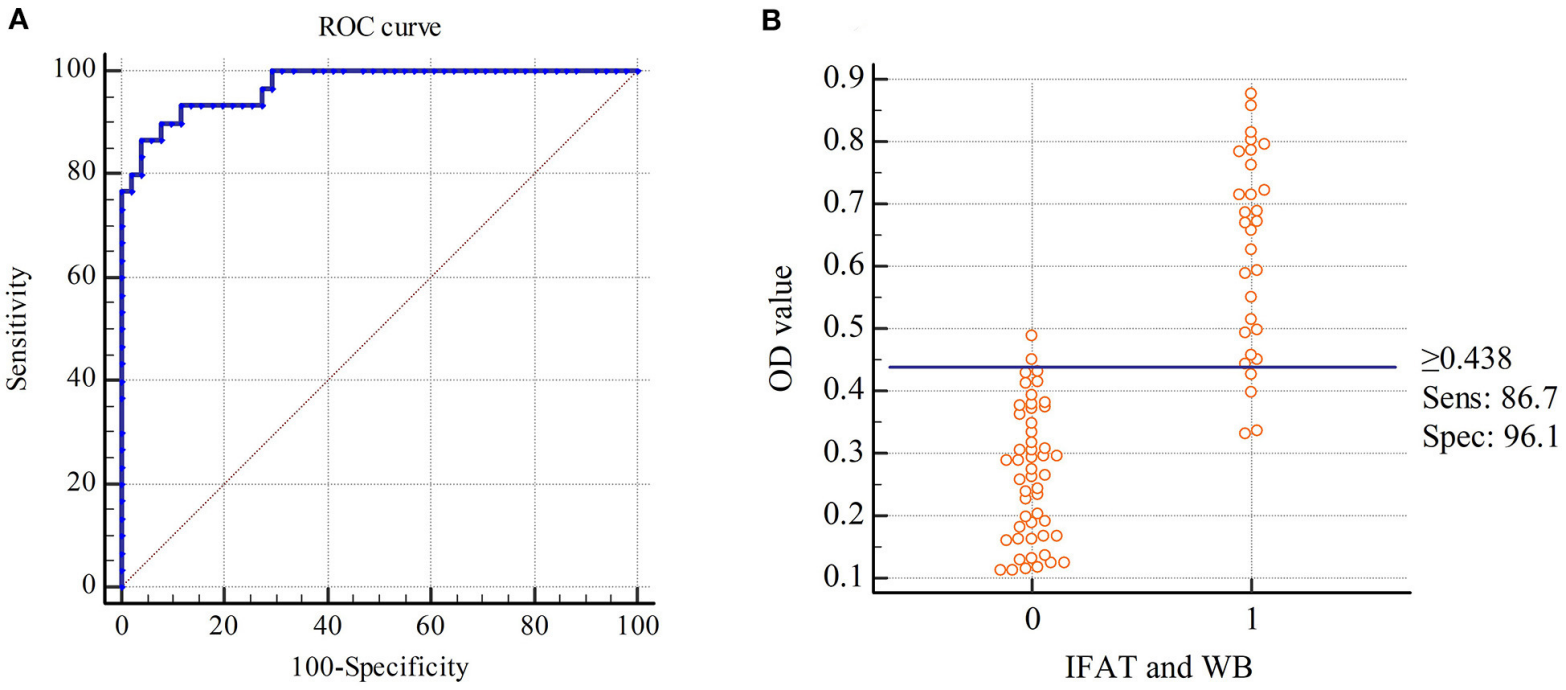
Receiver Operating Characteristics (ROC) analysis for the indirect ELISA with chimeric protein rSRS2-SAG1-GRA7. **(A)** ROC plot. Area under curve = 0.971 (0.0155); 95% confidence interval between 0.908 and 0.996. **(B)** Frequency distribution of confirmed positive (1) and confirmed negative (0) sera. Samples were considered positive when cut-off values were ≥ 0.438 ELISA absorbance values.

This developed rSRS2-SAG1-GRA7 indirect ELISA was applied to 81 serum samples with varied *N. caninum* antibody status. In these samples, 30 of them were tested *N. caninum*-positive by IFAT and WB, 51 of them were negative. The rSRS2-SAG1-GRA7 indirect ELISA detected 28 *N. caninum*-positive samples, 53 *N. caninum*-negative samples. And there are 26 samples that have been detected by these three methods as positive samples, 49 samples as negative samples. Based on ROC analysis (Figure 3A), an ELISA OD value of 0.438 was chosen as the threshold to distinguish between positive and negative samples, yielding a specificity of 96.1%, a sensitivity of 86.7%, and a kappa-value of 0.84 (Table 2). Figure 3B shows the frequency distribution of the positive and negative samples tested by IFAT and WB. In summary, the overall coincidence rate of the rSRS2-SAG1-GRA7 indirect ELISA to IFAT and WB was 92.6% (true positives + true negatives/total samples).

**Table 2:** Sensitivity, specificity and kappa-value of ELISA compared to IFAT and WB.

	**IFAT and WB**		
	**No. positive**	**No. negative**	**Total**
**ELISA**			
No. positive	26	2	28
No. negative	4	49	53
Total	30	51	81
	Sensitivity 86.7%	Specificity 96.1%	Kappa-value 0.84

### 3.3. *N. caninum* antibody detection of field serum samples

Sero-epidemiological data of for *N. caninum* infections in cattle from Ningxia are presented in Table 3. The 329 sera samples were collected from 11 farms with an average herd of more than 500 cows in 4 cities and examined by the ELISA technique. The herd prevalence was 100% and the overall seroprevalence was around 41.64%. The *N. caninum* antibody positive rate in each farm ranged from 19.05 to 57.89%. As shown in Table 4, the seroprevalence varied in different age groups, ranging from 28.26 to 44.04%, with the highest prevalence of 44.04% in >4 years dairy cattle, followed by 2–4 years dairy cattle (43.33%), indicating that the seroprevalence increased with age.

**Table 3:** Examination of anti-*N. caninum* antibodies from field samples with ELISA in Ningxia.

**City**	**Codes of farm**	**No. of positive**	**No. of sera**	**Positive ratio (%)**
Shizuishan	A	9	25	36.00
Wuzhong	B	11	31	35.48
	C	15	29	51.72
	D	6	15	40.00
Yinchuan	E	30	56	53.57
	F	5	13	38.46
	G	30	85	35.29
	H	11	19	57.89
	I	4	21	19.05
	J	7	14	50.00
Zhongwei	K	9	21	42.86
Total		137	329	41.64

**Table 4:** Seroprevalence of *N. caninum* infection in different age groups of dairy cattle using ELISA in Ningxia of China.

**Age (yr)**	**No. examined**	**No. positive**	**Prevalence (%)**
≤ 2	46	13	28.26
2–4	90	39	43.33
> 4	193	85	44.04

## 4. Discussion

Screening and culling of infected cattle based on reliable diagnostic techniques is a strategy to eliminate or reduce infection in a herd. At present, indirect ELISA is commonly used in the epidemiological investigation of neosporosis. The main antigens used are surface antigen superfamily members SAG1 and SRS2. *N. caninum* bradyzoite antigen SAG4 is also used to detect chronic infection of neosporosis. Dense granule proteins GRA2, GRA7 and microneme protein are also serological diagnostic antigens of neosporosis (30, 31). The ELISA detection methods established by different antigens have great differences in the detection coincidence rate of samples from different countries (32). Here, we developed an indirect ELISA with a chimeric protein rSRS2-SAG1-GRA7 for the diagnosis of cattle neosporosis. The immunodominant surface proteins of *N. caninum*, NcSRS2, NcSAG1 and NcGRA7, were immobilized with linkers and the chimeric protein rSRS2-SAG1-GRA7 was expressed in the procaryotic expression system under standard conditions without needing any special requirements. No antigen-antibody reactions were detected in the western blotting performed with rSRS2-SAG1-GRA7 as antigen and sera from mouse infected with related apicomplexan parasite, *T. gondii*.

Several studies have described the development of ELISA using *N. caninum*-specific proteins, including NcSRS2, NcSAG1 and NcGRA7, for detecting the antibodies to *N. caninum* infection in animals. Borsuk et al. (14) described an indirect ELISA for cattle using NcSRS2 truncated protein and reported a sensitivity of 95% with 96% specificity (14). Then, a study also confirmed that the ELISA for sheep with the rNcSRS2 had 100% sensitivity and 94.5% specificity (33). Chahan et al. (34) described an indirect ELISA for cattle using NcSAG1 recombinant truncated protein, and the ELISA clearly differentiated between IFAT-positive and -negative sera from cattle (34). Hamidinejat et al. (35) developed the rNcGRA7-based indirect ELISA in cattle and water buffalo, sensitivities and specificities were 94.64 and 90.38% respectively using sera of cattle, but were 98.57 and 86.54% in the case of buffaloes respectively (35). More recently, an indirect ELISA based on rNcSRS2/rNcSAG1 was developed for the diagnosis of cattle and ovine neosporosis, and the sensitivity and specificity performed with the proteins NcSRS2 and NcSAG1 in combination had higher than alone (20). In this study, we obtained 86.7% sensitivity and higher specificity (96.1%) when using this chimeric protein, suggesting its applicability to the practical diagnosis of neosporosis.

In China, Xu et al. (36) first detected a positive antibody to neosporosis in yak herds in the Xinjiang region, which was an evidence to the existence of neosporosis in Chinese cattle herds (36). As the study of *N. caninum* has deepened, reports of neosporosis infected cattle are growing and suggest that neosporosis is epidemic in China (37). The sero-epidemiological investigation results of *N. caninum* in dairy cattle (regardless of abortion history) in Ningxia with commercial ELISA by Tan et al., Li et al. and Kang et al. were 8.93, 7.01 and 24.11% respectively (22–24). Cao et al. (25) reported that the seroprevalence of *N. caninum* in aborting dairy cattle by commercial ELISA kits was 30.23% in Wuzhong, Ningxia (25). In contrast, the seroprevalence of *N. caninum* in aborting dairy cattle in this study was higher (41.64%), which may be caused by the surge of cross-regional trade of dairy cows in recent years. The seroprevalence of *N. caninum* in aborting dairy cattle in Ningxia was higher than that of the cows (regardless of abortion history), indicating that *N. caninum* infection may be an important cause of abortion in dairy cattle in Ningxia.

An early study in the Netherlands showed equal seroprevalence across all age groups, suggesting that the infection had most likely been transmitted through vertical transmission (8). However, a Danish study revealed that sero-positivity increased with age (38). But, few previous studies have shown an association of seroprevalence with age in aborting dairy cattle. In this study, the seroprevalence in the aborting dairy cattle varied in different age groups and the seroprevalence was positively correlated with age. The risk of *N. caninum* infection may increase with age suggesting that horizontal transmission of *N. caninum* was particularly important in these herds.

## 5. Conclusion

An indirect ELISA employing rSRS2-SAG1-GRA7 of *N. caninum* was developed. The method showed high sensitivity and specificity, suggesting its applicability to the practical diagnosis of *N. caninum* infection. This study showed a high prevalence (41.64%) of neosporosis in aborting dairy cattle in Ningxia, China. Therefore, infection with *N. caninum* may be one of the important causes of cattle abortion in this region, hence, dairy farmers need to practice proper management and control strategies urgently.

## Data availability statement

The datasets presented in this study can be found in online repositories. The names of the repository/repositories and accession number(s) can be found below: https://www.ncbi.nlm.nih.gov/genbank/, AF061249, AF132217, and AF176649&.

## Ethics statement

The animal study was reviewed and approved by Institutional Animal Care and Use Committee of Anhui Agricultural University (No. AHAU2020020). Written informed consent was obtained from the owners for the participation of their animals in this study.

## Author contributions

C-SY, C-YY, and Q-MX designed the study. C-YY, J-YZ, Y-HH, Y-QC, Z-XL, and H-XZ performed, collected data from experiment, and analyzed data. C-SY, C-YY, O-OA, Y-QC, Z-XL, L-YL, R-RC, XZ, and Q-QZ wrote the manuscript. All authors read and approved the final manuscript.

## References

[1] Donahoe SL Lindsay SA Krockenberger M Phalen D Šlapeta J. A review of neosporosis and pathologic findings of *Neospora caninum* infection in wildlife. Int J Parasitol Parasites Wildl. (2015) 4:216–38. 10.1016/j.ijppaw.2015.04.002 25973393 PMC4427759

[2] Bjerkas I Mohn SF Presthus J. Unidentified cyst-forming sporozoon causing encephalomyelitis and myositis in dogs. Z Parasitenkd. (1984) 70:271–74. 10.1007/BF00942230 6426185

[3] Dubey JP. Neosporosis in cattle: biology and economic impact. J Am Vet Met Assoc. (1999) 214:1160–3. 10212674

[4] Deng C Zhang W Liu Q Hu DM Yu XL Liu J. Identification of *Neospora caninum* in abortion fetus of dairy cow in China. Chin Vet Sci. (2007) 37:16–9. 10.16656/j.issn.1673-4696.2007.01.004

[5] Ding D Jia LJ Tian WN Liang WF Zhang XC Zhang SF. Isolation and identification of Jilin strain *Neospora caninum*. Chin J Vet Sci. (2009) 29:423–5. 10.16303/j.cnki.1005-4545.2009.04.00925678924

[6] Reiterová K Spilovská S Antolová D Dubinský P. *Neospora caninum*, potential cause of abortions in dairy cows: the current serological follow-up in Slovakia. Vet Parasitol. (2009) 159:1–6. 10.1016/j.vetpar.2008.10.008 19019551

[7] Dubey JP Schares G Ortega-Mora LM. Epidemiology and control of neosporosis and *Neospora caninum*. Clin Microbiol Rev. (2007) 20:323–67. 10.1128/CMR.00031-06 17428888 PMC1865591

[8] Wouda W Bartels CJ Moen AR. Characteristics of *Neospora caninum*-associated abortion storms in diary herds in the Netherlands (1995 to 1997). Theriogenology. (1999) 52:233–45. 10.1016/S0093-691X(99)00125-9 10734391

[9] Lassen B Orro T Aleksejev A Raaperi K Järvis T Viltrop A. *Neospora caninum* in Estonian dairy herds in relation to herd size, reproduction parameters, bovine virus diarrhoea virus, and bovine herpes virus 1. Vet Parasitol. (2012) 190:43–50. 10.1016/j.vetpar.2012.05.021 22721941

[10] Malaguti JM Cabral AD Abdalla RP Salgueiro YO Galleti NT Okuda LH. *Neospora caninum* as causative agent of bovine encephalitis in Brazil. Rev Bras Parasitol Vet. (2012) 21:48–54. 10.1590/S1984-29612012000100010 22534945

[11] Dubey JP. Review of *Neospora caninum* and neosporosis in animals. Korean J Parasitol. (2003) 41:1–16. 10.3347/kjp.2003.41.1.1 12666725 PMC2717477

[12] Alvarez-García G García-Culebras A Gutiérrez-Expósito D Navarro-Lozano V Pastor-Fernández I Ortega-Mora LM. Serological diagnosis of bovine neosporosis: a comparative study of commercially available ELISA tests. Vet Parasitol. (2013) 198:85–95. 10.1016/j.vetpar.2013.07.033 23953144

[13] Sinnott FA Monte LG Collares TF Silveira RM Borsuk S. Review on the immunological and molecular diagnosis of neosporosis (years 2011–2016). Vet Parasitol. (2017) 239:19–25. 10.1016/j.vetpar.2017.04.008 28495191

[14] Borsuk S Andreotti R Leite FP Pinto Lda S Simionatto S Hartleben CP. Development of an indirect ELISA-NcSRS2 for detection of *Neospora caninum* antibodies in cattle. Vet Parasitol. (2011) 177:33–8. 10.1016/j.vetpar.2010.11.026 21168278

[15] Hosseininejad M Hosseini F Mosharraf M Shahbaz S Mahzounieh M Schares G. Development of an indirect ELISA test using an affinity purified surface antigen (P38) for sero-diagnosis of canine *Neospora caninum* infection. Vet Parasitol. (2010) 171:337–42. 10.1016/j.vetpar.2010.04.003 20434268

[16] Jenkins M Parker C Tuo W Vinyard B Dubey JP. Inclusion of CpG adjuvant with plasmid DNA coding for NcGRA7 improves protection against congenital neosporosis. Infect Immun. (2004) 72:1817–9. 10.1128/IAI.72.3.1817-1819.2004 14977994 PMC356056

[17] Dong J Otsuki T Kato T Kohsaka T Ike K Park EY. Development of two murine antibodies against *Neospora caninum* using phage display technology and application on the detection of *N. caninum*. PLoS ONE. (2013) 8:e53264. 10.1371/journal.pone.0053264 23308179 PMC3540087

[18] Howe DK Crawford AC Lindsay D Sibley LD. The p29 and p35 immunodominant antigens of *Neospora caninum* tachyzoites are homologous to the family of surface antigens of *Toxoplasma gondii*. Infect Immun. (1998) 66:5322–8. 10.1128/IAI.66.11.5322-5328.1998 9784539 PMC108665

[19] Liddell S Lally NC Jenkins MC Dubey JP. Isolation of the cDNA encoding a dense granule associated antigen (NCDG2) of *Neospora caninum*. Mol Biochem Parasitol. (1998) 93:153–8. 10.1016/S0166-6851(98)00031-0 9662039

[20] Alves Sinnott F da Silva Leal K de Oliveira Silva MT Barros de Pinho R Pappen F da Rosa Farias NA. An indirect ELISA for Neosporosis: associating recombinant Neospora caninum proteins NcSRS2 and NcSAG1 Vet Parasitol. (2020) 281:109101. 10.1016/j.vetpar.2020.109101 32302944

[21] Yang C Liu J Ma L Zhang X Zhang X Zhou B. NcGRA17 is an important regulator of parasitophorous vacuole morphology and pathogenicity of *Neospora caninum Vet Parasitol*. (2018) 264:26–34. 10.1016/j.vetpar.2018.03.018 30503087

[22] Tan QD Wei BX Chen B Zhang XX Yang YC Qin SY. Seroprevalence and risk factors of *Neospora caninum* in dairy cattle in Gansu and Ningxia. Chin J Anim Infect Dis. (2016) 24:46–50.

[23] Li Z Zhao Y Li J Song QR Li ZX. Serological investigation of neosporosis in dairy cows in large-scale in Ningxia. Gansu Anim Husbandry Vet. (2017) 47:61–3. 10.15979/j.cnki.cn62-1064/s.2017.02.020

[24] Kang XD Gao HH Tuo ZJ Shao HF Zhang PH Ma ZM. Epidemiological investigation of neosporosis in dairy cows in Ningxia area. Zhongguo Xumu ShouYi Wenzai. (2017) 33:111.

[25] Cao S Guo Y Lei Y Bai X Ma Y Yu Y. Serological investigation on infectious pathogens in abortion cows of large-scale dairy farms in Wuzhong of Ningxia. Pro Vet Med. (2016) 37:115–9.

[26] Gao Y Guo H Moumouni PF Liu M Li J Efstratiou A. Development and evaluation of an enzyme-linked immunosorbent assay based on recombinant TgSRS2 for serodiagnosis of *Toxoplasma gondii* infection in cats. J Vet Med Sci. (2020) 82:1662–65. 10.1292/jvms.20-0231 33071252 PMC7719894

[27] Schares G Rauser M Zimmer K Peters M Wurm R Dubey JP. Serological differences in *Neospora caninum*-associated epidemic andendemic abortions. J Parasitol. (1999) 85:688–94. 10.2307/3285744 10461950

[28] Paré J Hietala SK Thurmond MC. Interpretation of an indirect fluorescent antibody test for diagnosis of *Neospora* sp. infection in cattle. J Vet Diagn Invest. (1995) 7:273–5. 10.1177/104063879500700222 7619917

[29] Fernandes BC Gennari SM Souza SL Carvalho JM Oliveira WG Cury MC. Prevalence of anti-*Neospora caninum* antibodies in dogs from urban, periurban and rural areas of the city of Uberlândia, Minas Gerais–Brazil. Vet Parasitol. (2004) 123:33–40. 10.1016/j.vetpar.2004.05.016 15265569

[30] He P Li J Gong P Liu C Zhang G Yang J. *Neospora caninum* surface antigen (p40) is a potential diagnostic marker for cattle neosporosis. Parasitol Res. (2013) 112:2117–20. 10.1007/s00436-013-3309-3 23435920

[31] Liu J Yu JS Liu Q Wang M. Establishment of recombinant dNcSRS2 protein based indirect ELISA for detection of antibody against *Neospora caninum* and its application. Chin J Anim Vet Sci. (2006) 37:1036–41.

[32] Yin J Qu G Cao L Li Q Fetterer R Feng X. Characterization of *Neospora caninum* microneme protein 10 (NcMIC10) and its potential use as a diagnostic marker for neosporosis. Vet Parasitol. (2012) 187:28–35. 10.1016/j.vetpar.2012.01.003 22284302

[33] Pinheiro AF Borsuk S Berne ME Pinto Lda S Andreotti R Roos T. Use of ELISA based on NcSRS2 of *Neospora caninum* expressed in Pichia pastoris for diagnosing neosporosis in sheep and dogs. Rev Bras Parasitol Vet. (2015) 24:148–54. 10.1590/S1984-29612015015 26083692

[34] Chahan B Gaturaga I Huang X Liao M Fukumoto S Hirata H. Serodiagnosis of *Neospora caninum* infection in cattle by enzyme-linked immunosorbent assay with recombinant truncated NcSAG1. Vet Parasitol. (2003) 118:177–85. 10.1016/j.vetpar.2003.10.010 14729165

[35] Hamidinejat H Seifi Abad Shapouri MR Namavari MM Shayan P Kefayat M. Development of an Indirect ELISA Using Different Fragments of Recombinant Ncgra7 for Detection of *Neospora caninum* Infection in Cattle and Water Buffalo. Iran J Parasitol. (2015) 10:69–77. 25904948 PMC4403542

[36] Xu XP Cheng ZR Bo XW. Dieter Palmer. Serological investigation of neosporosis in some parts of Xingjiang. Chin Vet Sci. (2002) 32:25–6. 10.16656/j.issn.1673-4696.2002.05.014

[37] Han FJ Liu J Nan HZ Liu Q. Serological investigation of neosporosis in a cattle farm in Beijing. Chin J Vet Med. (2015) 51:50–2.

[38] Jensen AM Björkman C Kjeldsen AM Wedderkopp A Willadsen C Uggla A. Associations of *Neospora caninum* seropositivity with gestation number and pregnancy outcome in Danish dairy herds. Prev Vet Med. (1999) 40:151–63. 10.1016/S0167-5877(99)00048-3 10423771

